# Supplementary material for: Longitudinal Imaging Using PET/CT with Collagen-I PET-Tracer and MRI for Assessment of Fibrotic and Inflammatory Lesions in a Rat Lung Injury Model
Source: J Clin Med. 2020 Nov 18;9(11):3706. doi: 10.3390/jcm9113706 (PMC7699272; doi:10.3390/jcm9113706)
Supplement: Supplementary file 1 [file jcm-09-03706-s001.pdf]

**Supplementary files for**  
**Longitudinal imaging using PET/CT with type I collagen PET-tracer**  
**combined with MRI for assessment of fibrotic and inflammatory lesions in**  
**a rat model of lung injury induced by bleomycin**

Irma Mahmutovic Persson\*, Nina Fransén Pettersson, Jian Liu, Hanna Falk Håkansson,  
Anders Örbom, René In't Zandt, Ritha Gidlöf, Marie Sydoff, Karin von Wachenfeldt and  
Lars E. Olsson - on behalf of the TRISTAN Consortium

\* Corresponding author E-mail: [irma.mahmutovic\\_persson@med.lu.se](mailto:irma.mahmutovic_persson@med.lu.se)

Supplementary data files:

- 1) Material and Methods section of imaging parameters
  - (I) MRI
  - (II) PET/CT
- 2) Material and Methods section of the radiochemistry procedure.
- 3) Pilot study description and results of
  - (I) Organ distribution of tracer during live imaging,
  - (II) Organ distribution of tracer ex vivo.
- 4) Imaging data analysis and Lung-ROI definitions.
- 5) Forward and Reverse Primer sequences from the gene expression analysis (Table).
- 6) Additional Supplementary Figures (Figure S1-S3).

## ***Supplementary file 1:***

### ***I) Magnetic Resonance Imaging***

Preclinical MRI system: 9.4T MRI Biospec AV III (Bruker, Ettlingen, Germany)

Software for operating MRI: ParaVision 6.0.1 (Bruker).

Protocol: a) Localizer for positioning of FOV b) UTE ( $TE_{\text{SHORT}}$ ) with 0.324 ms, c) TE ( $TE_{\text{LONG}}$ ) with 1 ms.

Repetition time (TR): 8 ms

Flip angle: 25°

FOV: 58x58 mm<sup>2</sup>

Matrix size: 192x192

2D transverse slice thickness 2 mm

Slices: approximately a total of 19 slices were needed to cover a whole rat lung. (Minimum 18 slices and maximum 22.)

Image reconstruction: images were reconstructed and zero filled to a 256x256 matrix.

Reconstructed pixel resolution: 0.22x0.22 mm<sup>2</sup>.

RF coil: 86 mm diameter quadrature (Bruker, Ettlingen, Germany).

Triggering: No respiratory nor ECG trigger was used.

Image acquisition: a total of 4 averages were used to reduce motion artefacts.

Total MRI experimental time: was  $25 \pm 5$  min in total.

Preparation of imaging system was done to improve overall image quality by measuring the trajectory of the radial acquisition on a phantom, using the experimental in vivo settings for the UTE sequence.

## *II) Positron Emission Tomography-Computed Tomography (PET-CT)*

Used CT scan parameters were:

Power: 65 kVp,

Exposure time: 500 ms,

Voxel size: 0.14x0.14x0.14 mm<sup>3</sup>.

Immediately after CT imaging, a PET scan was performed.

Post-reconstruction of PET: protocol of MLEM (Maximum Likelihood Estimation Method) with 4 iterations and 6 subsets (Nucline™, Mediso, Hungary).

Voxel size after reconstruction: 0.4x0.4x0.4 mm<sup>3</sup>.

## *Supplementary file 2:*

### *Radiochemistry*

#### *A) Materials and Methods*

CBP-NODAGA was used as received on 2018-06-07 from P. Caravan (Harvard, Boston, USA). 3.0 M Sodium Acetate buffer, pH 4.5, and 3.0 M sodium hydroxide, were prepared in house from metal free water (Sigma), Glacial Acetic Acid (Merck) and Sodium Hydroxide (Merck) and sterile filtered (Millex-GV, 0.22  $\mu\text{m}$ ) on the morning before use.  $\text{Cu}^{64}$  was received from Hevesy Laboratoriet, DTU Nutech Risø, Roskilde on the morning after production. Instant Thin Layer Chromatography (ITLC) strips were from Biodex, USA, Model# 150-771. Eluent for the ITLC was a 0.2 M Citric acid (Sigma Aldrich), also prepared in house. The radioactivity distribution of the ITLC strips were determined with a PhosphorImager system (Perkin Elmer, Waltham, MA, USA) using Optiquant as analysis software (Perkin Elmer). pH test strips were from Sigma, test range 0.0-6.0, P-4661, Lotnr: 010B164661.

The procedure received from P. Caravan was performed but due to retention of activity on the  $\text{C}^{18}$ -cartridge and the subsequent evaporation from ethanol was considered a too large drop in activity to perform the imaging study. In communications with Caravan the reason for using SepPak cartridges was to purify from residual Ge ions.<sup>1</sup> As the  $\text{Cu}^{64}$  is delivered with a specification of >99% purity as well as the ITLC analysis of the reaction proved the reaction to be higher yielding than 95%, it was deemed pure enough for a preclinical pilot study and the decision to inject the reaction solution as is after pH adjustment to 5.0 was made. The modified procedure was therefore changed according to discussion for adjusting the protocol without the use of SepPak.

#### *B) Storage of CBP-NODAGA*

The peptide was delivered to LBIC in December 2018 as a white powder. It was stored at -20°C. The vials were marked with 11/20/18, 0.61 mg, 8 doses and a vial number (#1-9) on delivery.

#### *C) Radiolabelling with $\text{Cu}^{64}$*

On the first radiolabelling of the week a vial containing CBP-NODAGA was thawed. To the vial was added 610  $\mu\text{L}$  of 3.0 M NaOAc (pH 4.5) and the vial was shaken until all powder was dissolved. 300  $\mu\text{L}$  of the solution was added directly into the vial containing  $\text{Cu}^{64}$ . The remaining CBP-NODAGA solution was refrozen to be used on the second labelling the same week. The reaction vial was swirled gently to rinse down any activity remaining on the walls. pH was analyzed and if below 4.0 it was adjusted with additional 3.0 M NaOAc (pH 4.5) until pH approximately 4.1 was obtained. After correct pH was achieved the vial was sealed with parafilm and heated for 20 min to 60°C. Labelling efficiency was monitored by applying 1  $\mu\text{L}$  to an ITLC strip and subsequent elution with 0.2 M citric acid. Radiolabelling yield was over 95% and solutions were used for injection and imaging without further purification. Note that these results prove that less than 5% of  $\text{Cu}^{64}$  is free in solution, this does not necessarily mean

---

that all peptide conjugates in the solution is labelled. After cooling for a few minutes the pH was adjusted to 4.5 using 3.0 M NaOH when needed, typically 40  $\mu$ L. The vial, containing 4 doses of peptide and 600-800 MBq radioactivity in approximately 340 $\mu$ L buffer was then sealed and delivered as is to the imaging lab. For the first 2 production days, the delivery day was the day after production and the procedure used a SepPak to further purify the product, but as the study resumed in October 2019 it was delivered immediately after analysis as described above.

#### *D) Analytical Results*

The ITLC strips were analyzed according to the following procedure: 1  $\mu$ L was applied to an ITLC strip and it was eluted in 0.2M Citric Acid. The point of origin contains the radioconjugate and anything eluted with the solvent front on the strip is free  $\text{Cu}^{64}$  according to figure 1. Figure 2 shows the elution profile for the labelled profile.

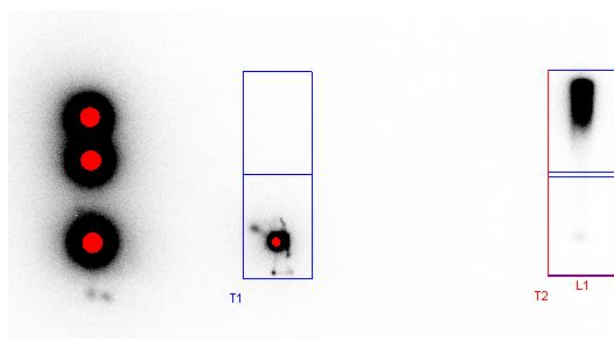

**Figure 1:** ITLC strips from 2019-10-24; furthest to the left a reference strip, middle (T1) 1  $\mu$ L of reaction mixture after heating. Right (T2) 1  $\mu$ L of  $\text{Cu}^{64}$ -solution before reaction in 3.0M NaOAc.

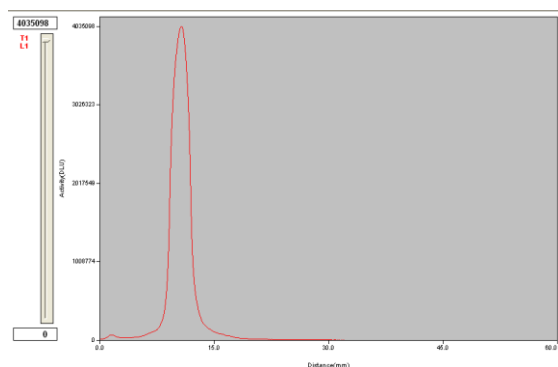

**Figure 2:** Elution profile of T1. 0.0-25.0 is considered origin, 35.0-55.0 is eluted material, i.e. free  $\text{Cu}^{64}$ .

Radiolabelling yields were over 95% for Cu<sup>64</sup>-CBP-NODAGA. The radiochemistry results are presented in Table 1.

| Date       | Free Cu <sup>64</sup> | Labelled product | Vial number |
|------------|-----------------------|------------------|-------------|
| 2019-10-15 | 1.5%                  | 98.5%            | 11/20/18#3  |
| 2019-10-17 | 3.3%                  | 96.7%            | 11/20/18#3  |
| 2019-10-22 | 1.3%                  | 98.7%            | 11/20/18#4  |
| 2019-10-24 | 1.3%                  | 98.7%            | 11/20/18#4  |
| 2019-10-29 | 0.6%                  | 99.4%            | 11/20/18#5  |
| 2019-10-31 | 1.2%                  | 98.8%            | 11/20/18#5  |
| 2019-11-05 | 1.4%                  | 98.6%            | 11/20/18#6  |
| 2019-11-07 | 1.6%                  | 98.4%            | 11/20/18#6  |

**Table 1:** *Presenting the analytical results from all the productions delivered for imaging.*

## Stability

To ensure stability of the radiolabelled peptide a stability study was performed on the first radiolabelled product from 2019-04-02. The product solution was injected onto a 1260 Agilent HPLC, equipped with a  $\mu$ BondapakC-18 column. Eluent was MeCN/H<sub>2</sub>O with 1% TFA gradient system.

As can be seen from the radiosignal chromatograms on the following page the readout from the radioactivity did not change in respect to retention time during the 30 hours it was monitored making it reasonable to believe that the tracer solution was stable before in vivo administration. This result is accordance with the stability of the previous labelling stability with Ga<sup>68</sup> performed with ITLC.

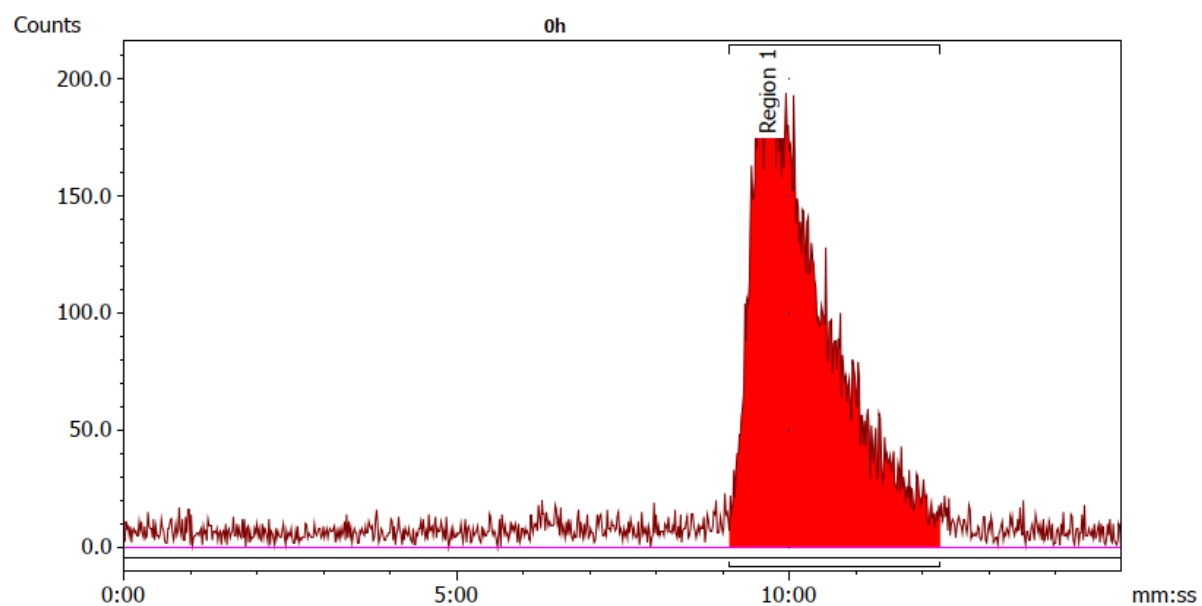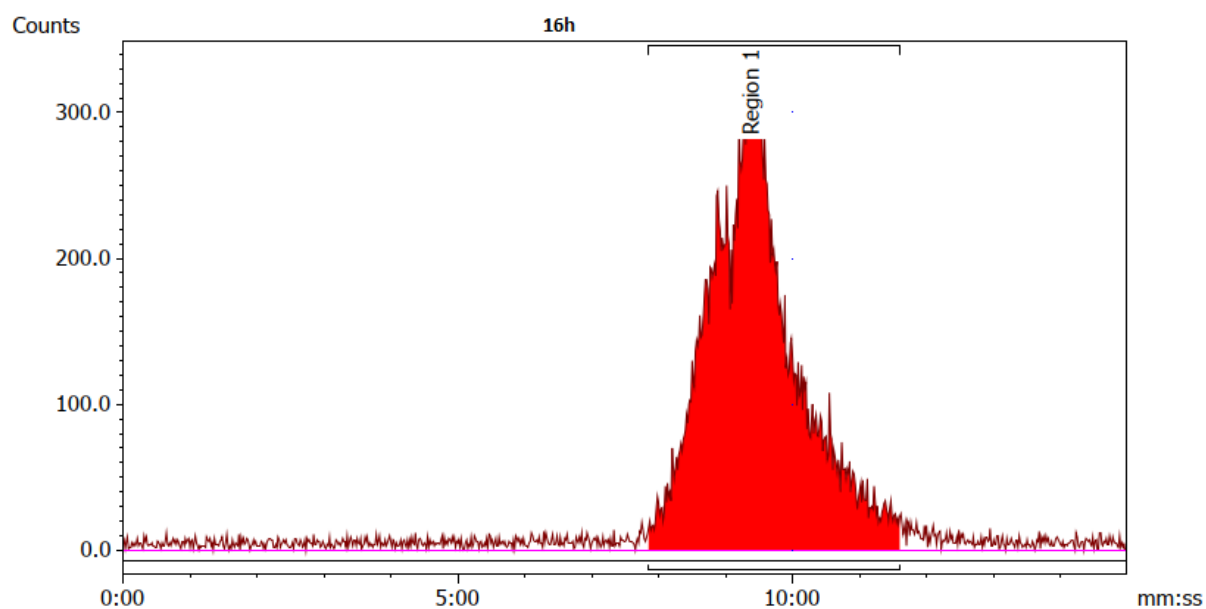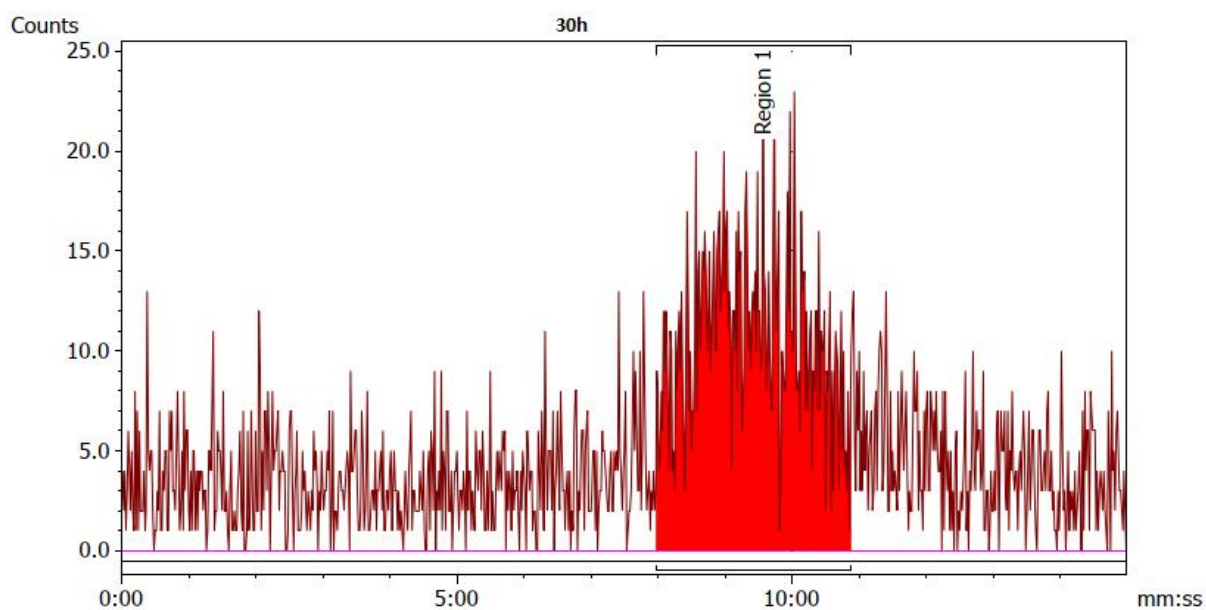

## Urine sample

A urine sample was obtained after the first imaging session (2019-04-02) which was analyzed on the same HPLC setup as previously mentioned on the day after the imaging study. As can be seen from the chromatograms below there is either a new adduct or free ions of  $\text{Cu}^{64}$  shown as a peak at retention time 4.2 min. Even the day after urine collection there is also a significant amount of  $\text{Cu}^{64}$ -CBP-NODAGA, retention time at 9.5 min. The ratio between the two is 68:32.

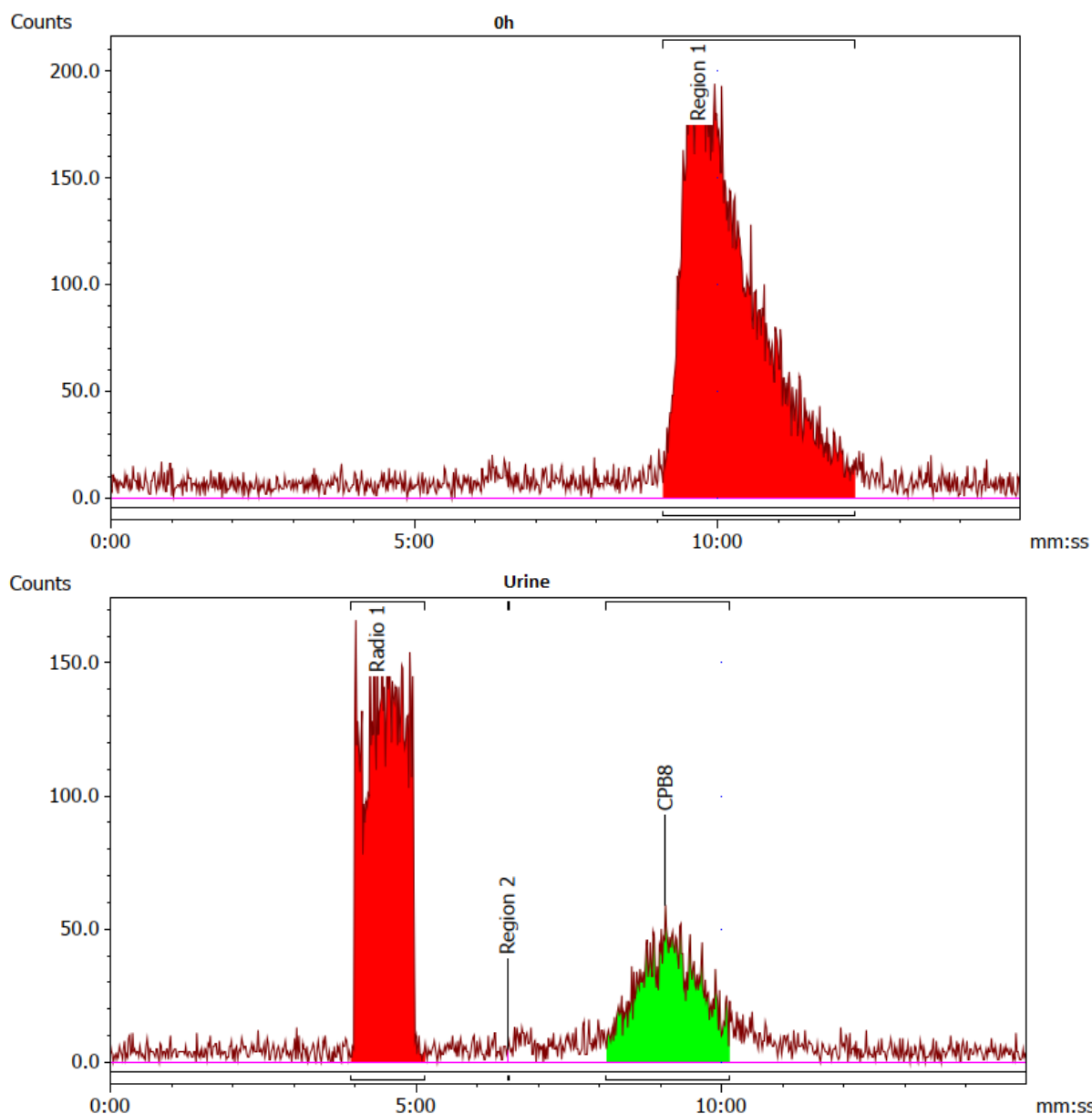

### *Supplementary file 3:*

#### *I) Organ distribution of tracer during live imaging*

A pilot study was performed before the main study. Below in Figure 1, three examples of rats are presented from tracer injections and PET signal detection. This was mainly done for evaluation of approximate organ distribution at 1-24h post-injection. Both bleomycin challenged rats and Saline control were injected with the PET-tracer and were imaged at different time points. The kidney and liver showed marked high signal up-take at all time points.

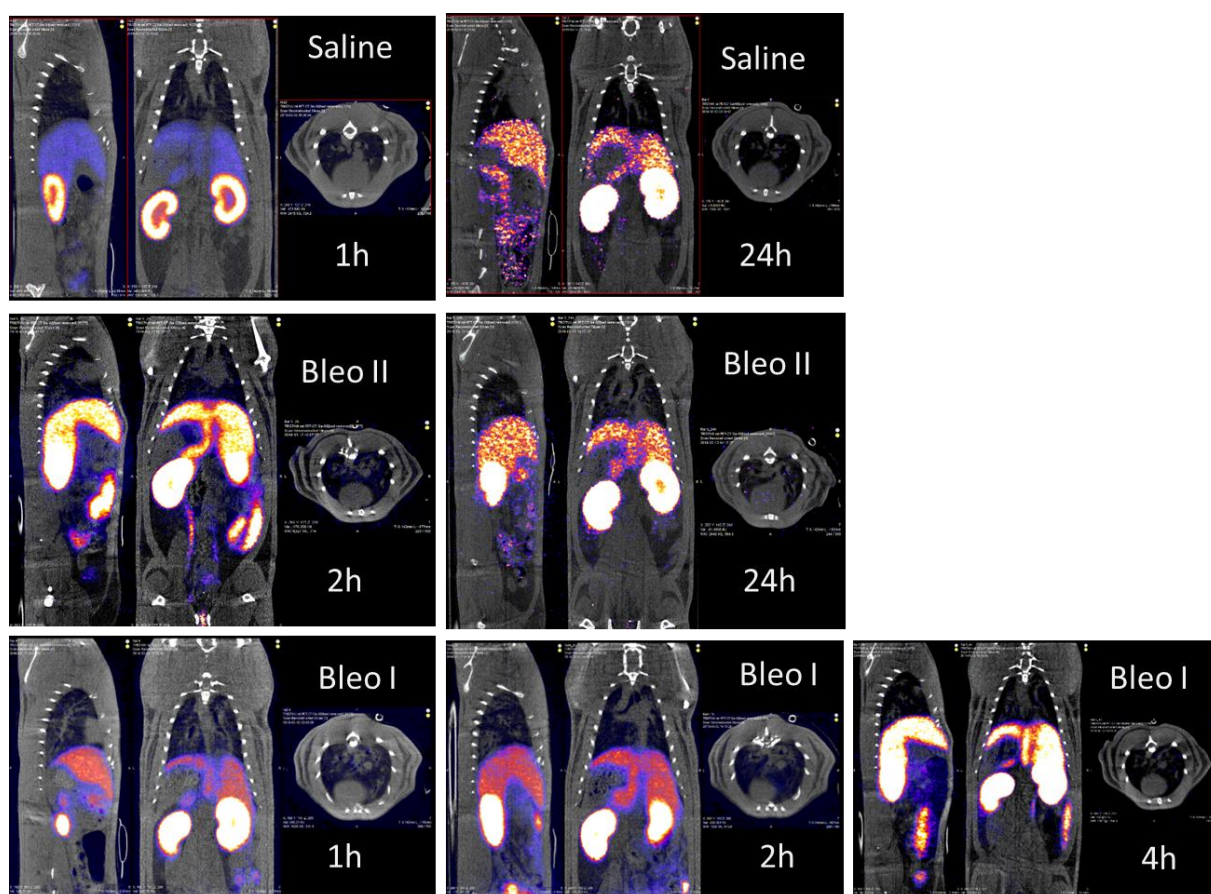

Figure 1: Images from PET overlaid on CT images. Organs distribution of the CBP-Cu<sup>64</sup> tracer during live imaging of one saline-challenged rat and two bleomycin-challenged rats, here shown in the coronal, sagittal and transverse plane, with all internal organs present within the FOV.

### Supplementary file 3:

#### II) Organ distribution of tracer *ex vivo*

Organs were collected 24h post-injection, after the last PET imaging, weight was noted and counts measured on an Automatic Gamma counter (WIZARD® 1480 RiaCalc WIZ, PerkinElmer). There is a marked high up-take in the liver and the kidneys. However, the difference between the Saline and Bleomycin-challenged rats clearly differ when comparing the lung up-take.

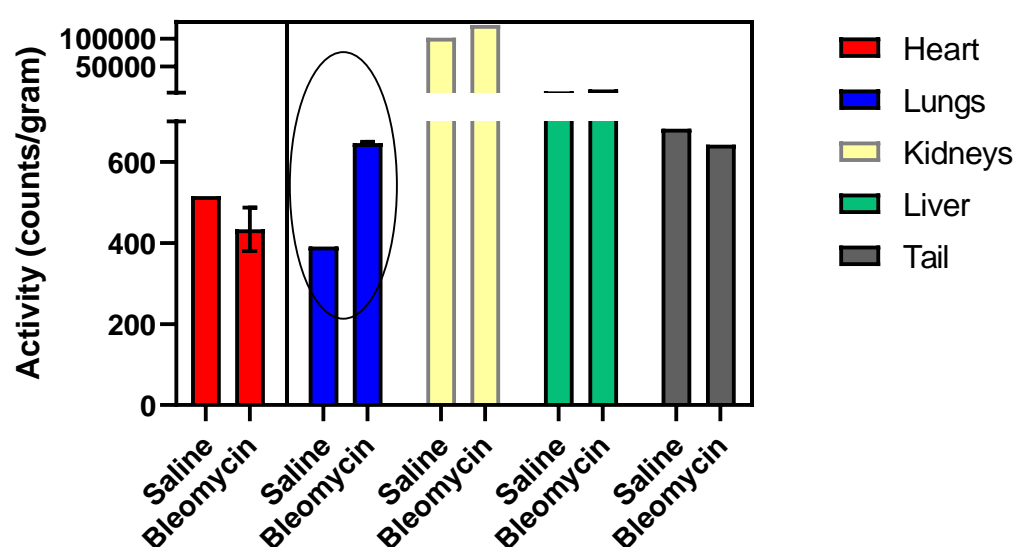

Figure 2: Organs distribution of the CBP-Cu<sup>64</sup> tracer, collected 24h post-injection and measured 2 days later (3 days after the injection). Activity was normalised to the injected dose, and decay corrected and presented per gram tissue for each organ.

#### ***Supplementary file 4:***

##### ***Image analysis from MRI scans***

The semi manually drawn regions of interest (ROI) were defined according to a template in each MRI slice, in a healthy rat, and followed as a map for all following scans. Example of the template ROI in three various slice positions are presented in Figure 1.

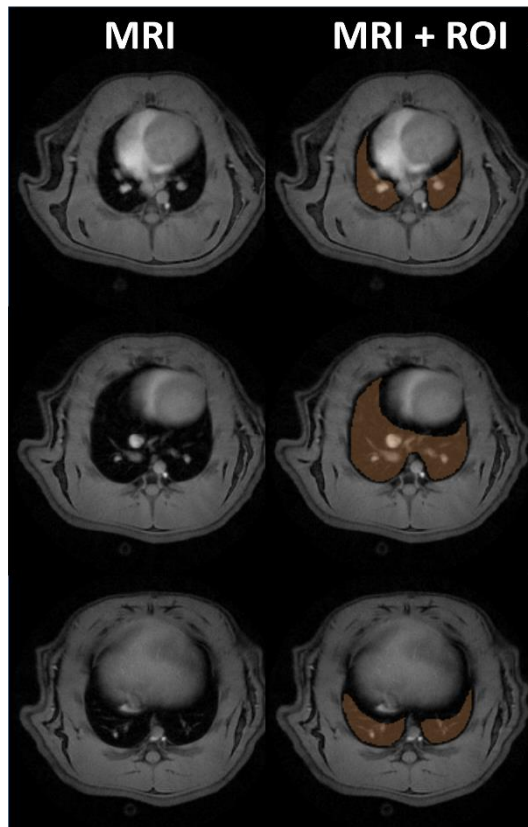

Figure 1: ROI template followed during the segmentation process of all scans. Drawn ROI is expressed in gold as a second layer on top of the original MRI image.

Once each ROI was completed within one animal scan, histograms of the data contained in the ROI were exported for both  $TE_{SHORT}$  and  $TE_{LONG}$ . The MRI histograms were batch analyzed using custom software written for the purpose in IDL 8.5 (Exelis VIS, Harris Corporation, Boulder, Colorado, USA). The software identifies the first peak in each histogram, representing the low-signal voxels in the normal lung. The point on the slope at the right side of this peak with the sharpest decline is then found and a linear function tangential to the slope is calculated. The sum of the volume in the histogram with signal above where the linear function intersects with the x-axis was defined as the MRI “high-signal” volume for each histogram, as shown in Supplementary Figure below.

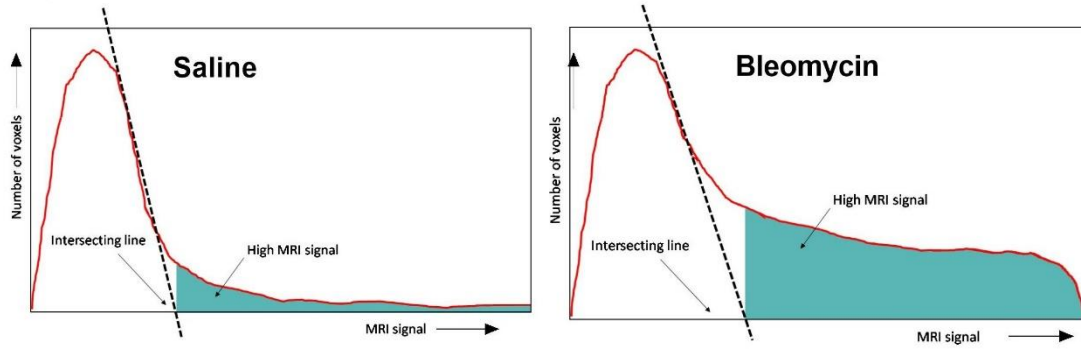

Figure 2: Histogram generated from one healthy lung region (Saline) and one Bleomycin-challenged rat, indicating the MRI signal in relation to the amount of voxels. High-signal voxels consist of signal from the vessels (alone in healthy lungs) and also lesions (besides the vessels in Bleomycin rats).

Quantification of the signal within the lung-ROI was done according to histogram analysis and threshold-based assessment in order to define the so called high-signal region within the lung-region. The two different TE were assessed for high-signal and the volume of these regions were acquired. Once defining the regions for  $TE_{LONG}$  and subtracting the regions representing the vessels (as only high-signal in the control animals), the “fluid” signal was quantified, representing the inflammation and edema within the lung region. The “tissue” signal was quantified by a subtraction of the  $TE_{LONG}$  image (fluid and vessels) from the  $TE_{SHORT}$  image (fluid, tissue and vessels). Thereby the fluid signal as well as signal from vessels are removed and only tissue remains

### *Image analysis from PET-CT scans*

The MRI data as well as the ROI was resampled into isotropic voxels before the PET and CT scans of the same animal, at the same time point, were imported into the software session. To match these disparate datasets, MRI and ROI were used as reference while an automatic registration was performed of PET and CT datasets to the MRI datasets. The settings of Rigid translation, and Fine quality, was employed in VivoQuant<sup>TM</sup> 3.5 which uses a mutual information metric to fit the datasets together using translations and rotations during a maximum of 200 iterations. Post registration, the activity distribution in the PET image within the lung ROI was measured, both as activity uptake per  $mm^3$  and total uptake in the whole lung, and divided by the decay-corrected injected dose per animal to find the fractional uptake. Subsequently, by using the histogram based analysis of the drawn total lung region, the “high-signal” from the MR-images were expressed. The high-signal areas from  $TE_{LONG}$  and  $TE_{SHORT}$  were then segmented as lesion-areas and PET signal was assessed within these regions as well as outside (e.g. health appearing lung areas).

***Supplementary file 5:***

*Table 1: Primer sequences Forward and Reverse from gene expression analysis.*

| No of Primer | Gene              | Product name | Sequence_forward      | Sequence_reverse       |
|--------------|-------------------|--------------|-----------------------|------------------------|
| 1            | TGFβ <sub>1</sub> | TGFb1        | GGAAATCAATGGGATCAGTC  | CTGAAGCAGTAGTTGGTATC   |
| 2            | TGFβ <sub>2</sub> | TGFb2        | TACAATGCTAACTTCTGTGC  | AATGTAGTAGAGGATGGTCAG  |
| 3            | TGFβ <sub>3</sub> | TGFb3        | CTGTCCATGTCATACCTTTC  | TGTCCACTCCTTTGAATTTG   |
| 4            | Integrinβ6        | ITGb6        | TTCAGAAGGATTCTGGGAAC  | GTTGAAGGATGCTGTATCTC   |
| 5            | Gremlin           | GREM1        | CTGAGGTGACAGAATGAATC  | GACCCTTTCTTTTCCCTTC    |
| 6            | α-SMA             | ACTA1        | AAACCACCTATAACAGCATC  | AGACAGAATATTTGCGTTCTG  |
| 7            | Collagen I        | COL1a2       | AGTGGAAGAGCGATTACTAC  | ATTGATGGTCTCTCCTAACC   |
| 8            | Collagen III      | COL3a1       | TTTCAAGATCAACACTGAGG  | TATTCTCCGCTCTTGAGTTC   |
| 9            | Lysyl Oxidase     | LOX          | CACCGTATTAGAAAGAAGCTC | GAAAGAGTCCAAACAAAACC   |
| 10           | Periostin         | POSTN        | AGAAAGACATTGTGACGAAG  | ACTTGTTTAGCAGAATCAGG   |
| 11           | Serpine1 (PAI-1)  | SERPINE1     | CAATCCAACAGAGACAATCC  | GGTTGGAAAGATTTACCAGTG  |
| 12           | SerpinH1          | Serpine H1   | ATCAGTGTTTCATATTCGTGG | GAATCGATTTTAAGGGTGGG   |
| 13           | IL-1β             | IL1b         | TAAGCCAACAAGTGGTATTC  | AGGTATAGATTCTTCCCCTTG  |
| 14           | TIMP-1            | TIMP1        | TTGATAGCTTCCAGTAAAGC  | GAATCCTTTGAGCATCTTAGTC |
| 15           | MUC1              | Muc 1        | CGGAAGTCAATGTGAATGAG  | CAAAATACAGACCAGTACCAG  |

*Supplementary file 6:*

*Supplementary Figures from the Results section*

**Supplementary Figure S1:**

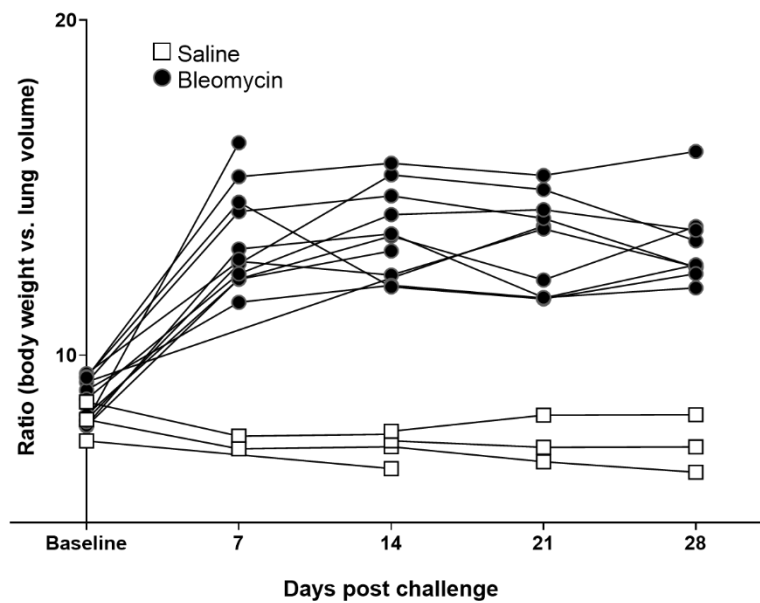

**Figure S1: Disproportional lung volume increase in bleomycin challenged rats.** Body weight to lung volume-ratio indicating disproportional total lung volume increase upon bleomycin challenge. Lung ratio was generated by calculating lung volume per body weight at that particular scan session, and presented as individual dots.

**Supplementary Figure S2:**

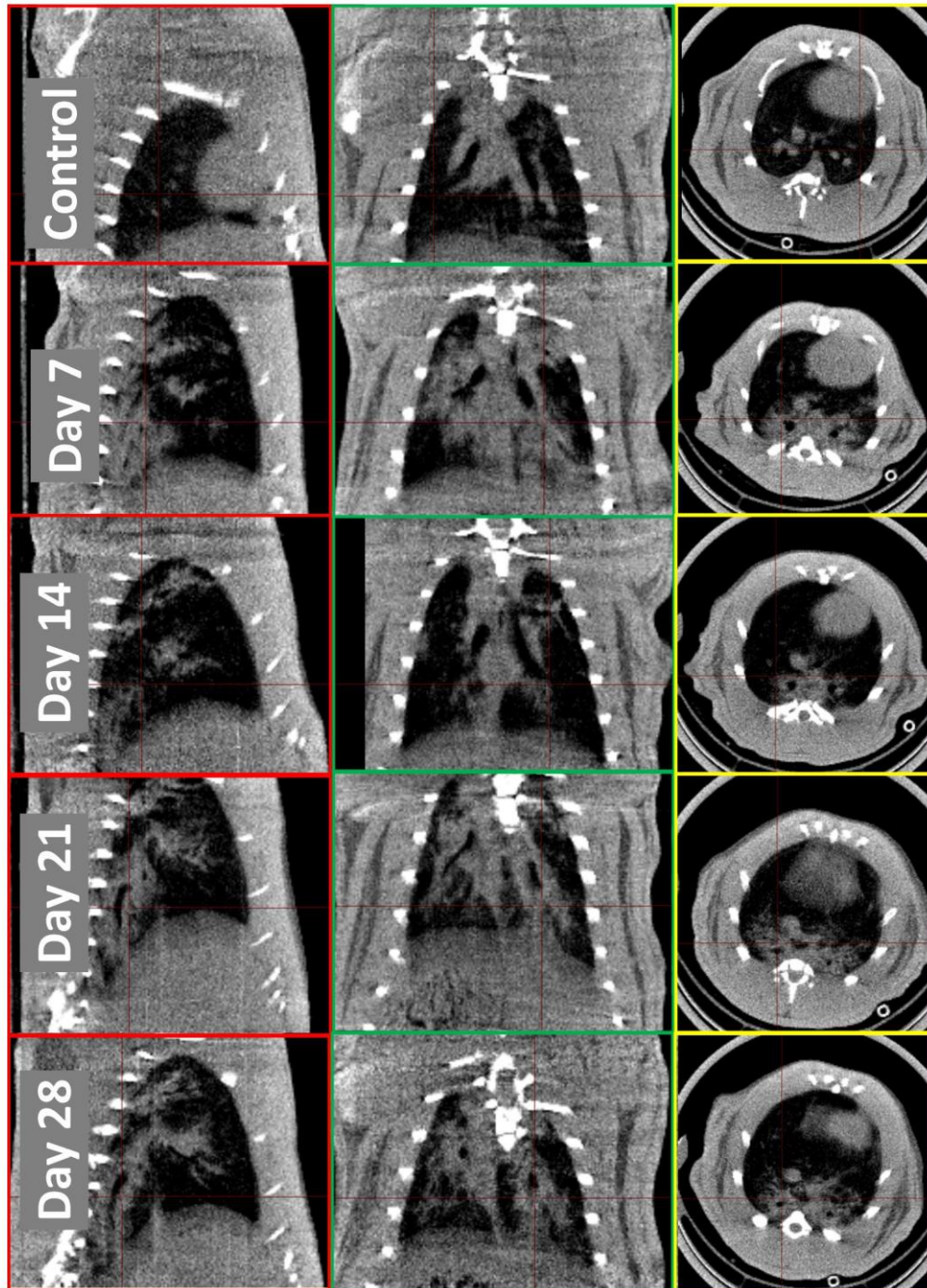

**Figure S2: Longitudinal computed tomography imaging in Group I, during 28 days of bleomycin-induced lung injury.** Representative CT images of lungs from rats challenged by i.t. administration of bleomycin (or saline as control) on day 7, 14, 21, and 28 post-challenge. The image views are presented from the sagittal (red frame), coronal (green frame) and axial (yellow) plane.

**Supplementary Figure S3:**

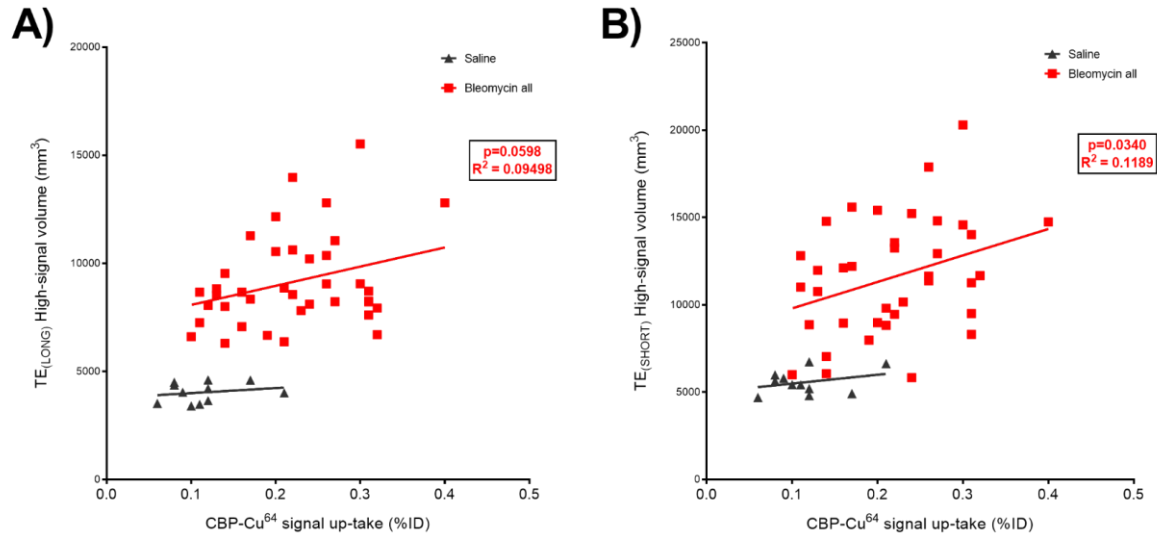

**Figure S3: Correlation of MRI signal vs. PET tracer up-take.** PET signal within the whole Lung-ROI was correlated to MRI signal acquired by the A) UTE sequence TE<sub>LONG</sub>, and B) UTE sequence TE<sub>SHORT</sub>.
